# Deep learning-based prediction of plant height and crown area of vegetable crops using LiDAR point cloud

**DOI:** 10.1038/s41598-024-65322-8

**Published:** 2024-06-28

**Authors:** Reji J, Rama Rao Nidamanuri

**Affiliations:** 1 Department of Earth and Space Sciences, Indian Institute of Space Science and Technology, Department of Space, Government of India, Thiruvananthapuram, 695 547 India; 2 School of Data Science, Indian Institute of Science Education and Research, Thiruvananthapuram, 695551 India

**Keywords:** Crop height, Crown area, Deep learning, LiDAR point cloud, Precision agriculture, Predictive modelling, Plant sciences, Engineering, Mathematics and computing

## Abstract

Remote sensing has been increasingly used in precision agriculture. Buoyed by the developments in the miniaturization of sensors and platforms, contemporary remote sensing offers data at resolutions finer enough to respond to within-farm variations. LiDAR point cloud, offers features amenable to modelling structural parameters of crops. Early prediction of crop growth parameters helps farmers and other stakeholders dynamically manage farming activities. The objective of this work is the development and application of a deep learning framework to predict plant-level crop height and crown area at different growth stages for vegetable crops. LiDAR point clouds were acquired using a terrestrial laser scanner on five dates during the growth cycles of tomato, eggplant and cabbage on the experimental research farms of the University of Agricultural Sciences, Bengaluru, India. We implemented a hybrid deep learning framework combining distinct features of long-term short memory (LSTM) and Gated Recurrent Unit (GRU) for the predictions of plant height and crown area. The predictions are validated with reference ground truth measurements. These predictions were validated against ground truth measurements. The findings demonstrate that plant-level structural parameters can be predicted well ahead of crop growth stages with around 80% accuracy. Notably, the LSTM and the GRU models exhibited limitations in capturing variations in structural parameters. Conversely, the hybrid model offered significantly improved predictions, particularly for crown area, with error rates for height prediction ranging from 5 to 12%, with deviations exhibiting a more balanced distribution between overestimation and underestimation This approach effectively captured the inherent temporal growth pattern of the crops, highlighting the potential of deep learning for precision agriculture applications. However, the prediction quality is relatively low at the advanced growth stage, closer to the harvest. In contrast, the prediction quality is stable across the three different crops. The results indicate the presence of a robust relationship between the features of the LiDAR point cloud and the auto-feature map of the deep learning methods adapted for plant-level crop structural characterization. This approach effectively captured the inherent temporal growth pattern of the crops, highlighting the potential of deep learning for precision agriculture applications.

## Introduction

Crop management practices responding to the intra-field variability in crops are vital for optimizing the costs associated with agriculture and ensuring agro-ecological benefits. Assessment of crops' health and growth needs time-sensitive agronomic inputs. Various governments and international multi-lateral agencies have been using remote sensing-based inputs on a routine basis for planning and decision making in food production, inventory, distribution, and market interventions^1–4^. At the local and functional farm level, high-resolution remote sensing has been used for classification^5^, species^6,7^ differentiating crops and weeds^8^ stress and disease detection^9,10^ etc. Employing ancillary datasets and reference sample measurements, studies estimating various structural and biophysical parameters have been reported extensively^11–14^.

Acquiring time-series remote sensing data, the biophysical status of crops at different growth phases has been assessed for various food and cash crops^15^. The nature of the methods employed determines the possibility and quality of estimation of crops' biophysical parameters^16^. UAVs, or Unmanned Aerial Vehicles, are remotely operated aircraft equipped with cameras and sensors. In agriculture, they are utilized for tasks such as crop monitoring and data collection through high-resolution aerial imagery. Recent studies have evaluated the use of time-series UAV-based multispectral imagery to predict crop yield at different growth stages at the field level using a range of deep-learning approaches^17,18^. Deep learning architectures, such as CNN-LSTM, ConvLSTM, and 3D-CNN, have been compared, with the 3D-CNN architecture outperforming the other two architectures with a coefficient of determination (R^2^) of 0.96^17,18^. Additionally, DeepCropNet (DCN), a deep learning architecture proposed by^19^, has been utilized for county-level corn yield estimation in the USA from 1981 to 2016 using multi-source remote sensing data, demonstrating the effectiveness of an attention-based LSTM architecture for capturing temporal features. Furthermore, recent advances in in-situ plant-level sensing systems, known as ‘high-throughput phenotyping platforms’ (HTPP), have enabled the modelling and prediction of a range of plant traits throughout the plant cycle. Deep learning-based models have been developed for predicting plant-level yield for Arabidopsis using spectral imagery from an HTPP, and fluorescence imagery has been used to predict dry plant mass and fruit number^20^.

Despite these advancements, most research related to the prediction of crop traits has concentrated on yield or crop growth stages at a broad level or for a single crop or group of plants^21–23^, highlighting a gap in predicting crop growth parameters at the farm level with different crops from an appropriate data and method perspective. A comprehensive literature review reveals a significant research gap, with existing works primarily focusing on predicting yield or growth stages of single crops or groups of plants, neglecting the prediction of broad-based plant parameters such as plant height and crown area. This gap highlights the necessity to address the prediction of crop growth parameters at the farm level across different crops from an appropriate data and method perspective.

This work addresses the critical need for early prediction of crop growth parameters to facilitate dynamic farming management. The objective of this study is to develop and apply a deep learning framework for predicting plant-level crop height and crown area at different growth stages specifically for vegetable crops. Predicting broad-based plant parameters such as plant height and crown area is crucial for laying the groundwork for developing ex-ante models of crop growth trajectories. Leveraging the explicit sensing nature of 3D laser scanners, particularly terrestrial laser scanners (TLS), point clouds can compute reference plant attributes. Deep learning (DL) methods offer promising computational-methodological frameworks for evaluating the possibility of using TLS point clouds to develop a prediction system for the ex-ante estimation of plant structural parameters. However, a pertinent research question remains: What is the potential of DL methods for predicting crop structural variables at different growth stages? This study addresses this question by developing a DL-based temporal framework to predict crop structural parameters.

## Materials and methods

### Study area

**Figure 1:**
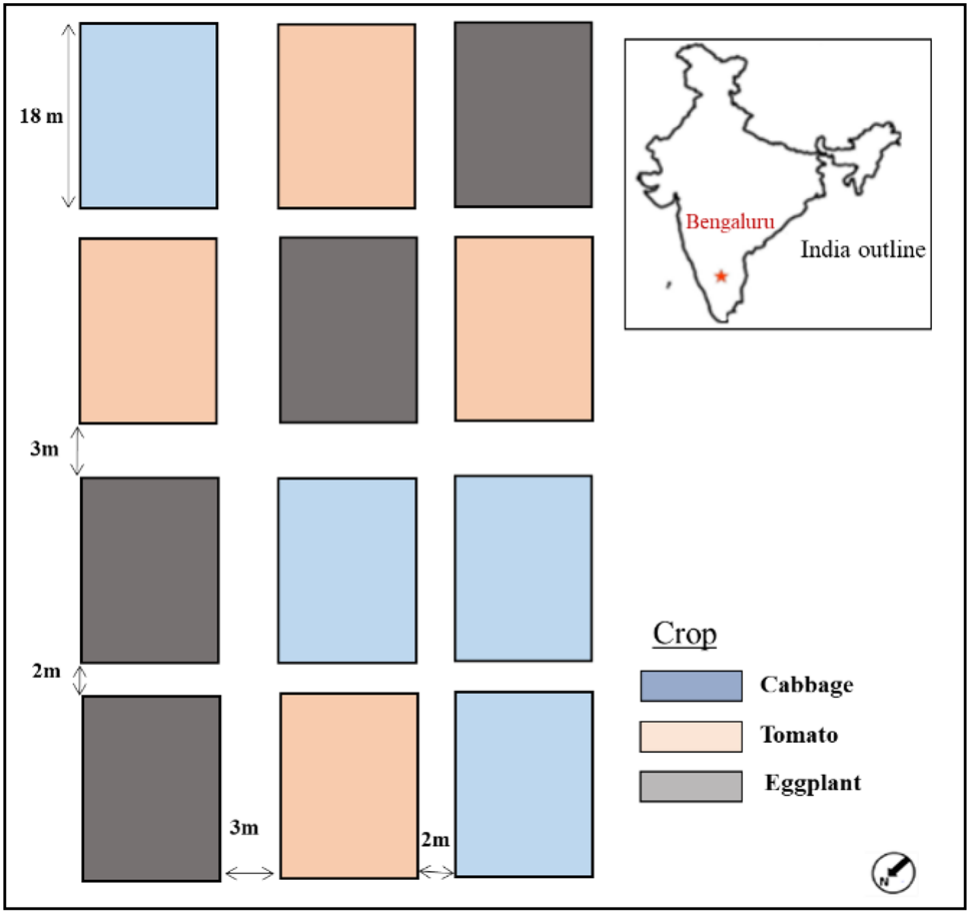
Location of Bengaluru in India and layout of the crop growing experiment.

In 2017 (January-May), a multi-crop drip-irrigated field experiment was established at the University of Agriculture Sciences (UAS), Bengaluru, India (Fig. 1). This experiment forms part of a larger project aiming to develop field-sensitive techniques using space technology and advanced pattern recognition for detecting crop type, growth condition, productivity, and ecosystem services. (Geographic coordinates:12°58′20.79''N, 77°34′50.31''E). Three different vegetable crops — tomato, eggplant, and cabbage — were cultivated in the field plots according to a factorial design. Thanks to its importance as a computer and electronics industry city, Bengaluru is one of the rapidly expanding cities in Asia and has been experiencing a host of transformations in land use and land cover conversions, and spatial distribution of grains, fruits, vegetables, and herbaceous crops. Geographically, Bengaluru lies off the Western Ghats of India, and the climate is mild, with a mean temperature of 29.2°C and annual precipitation of 873 mm.

### Experimental design and data acquisition

Based on the important regional food production and consumption patterns, three different vegetables – tomato, eggplant, and cabbage- were selected for growing on the experimental plots. Aligned with the onset of the Indian monsoon, the crop growing experiments were conducted during the Kharif (June to October) crop growing season. The cultivars selected for the crops correspond to those typically used by farmers across south India. All the experimental datasets on the plants cultivated were acquired complying to the mandatory institutional guidelines applicable.

The layout of the experimental set-up (Fig. 1) consisted of 12 plots, each of size 12 m × 18 m. There were four replications for each crop plot. To assess the nutrient responses and possible discrimination of crops with specific reference to nitrogen level, each of these plots were further divided into subplots of size 6 m × 12 m. Within each plot, three different levels of mineral nitrogen (N) fertilizer were supplied to the subplots randomly. Corresponding to the standard dose of the region and indicated as 'medium N' level in the experiment, urea at the rate of 46, 50, and 60 kg N ha^-1^ was applied to tomato, eggplant, and cabbage, respectively. The other two levels of nitrogen indicated as 'low N', and 'high N' correspond to the nitrogen application at the rate of 50% less and 50% more compared to the medium N level. Apart from the nitrogen, phosphorus (P) and potassium (K) were applied at sowing uniformly at the rate of 17.5 19.9, 16.6, and 41.5 kg K ha^-1^ for tomato, eggplant, and cabbage, respectively. The nitrogen was applied in two instalments to optimize the uptake and minimization of nutrients leaching due to heavy rainfall. In some of the plots, a moderate level of surface N transport occurred due to the heavy rainfall events during the crop growing experiments. As the primary aim of the work was predictive modelling of crop structural attributes, we have not considered the differential N rates in the experimental plots.

*Reference measurements of crop structural parameters*: Two different types of datasets were acquired over the experimental farms: LiDAR point cloud and reference crop structural parameters. Concurrent with the LiDAR point cloud acquisitions, reference measurements of plant height and crown area were obtained by distributing sampling locations across the plots. The plant height was measured using a ruler to the nearest centimetre. For each reference height measurement, the heights of 30 plants height were averaged, ensuring distribution in each subplot. Linear distance measurements of of plant crowns in the N-S and E-W directions were taken at several locations in the plots. The plant-level reference crown area was computed from the digital reconstruction of plants from the digital photographs as per^24^. The reference measurements were used as the training samples in the model development and to validate the predicted values.

**Figure 2:**
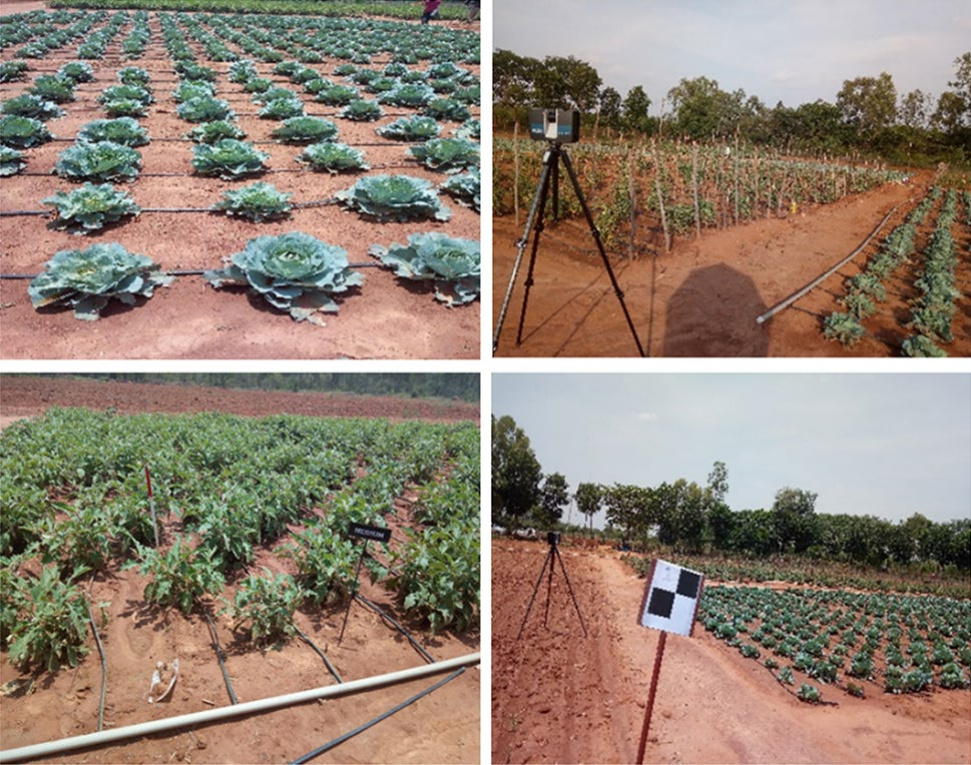
Field photographs showing the crops grown on the experimental plots and the positioning of the TLS and the reference targets.

**Figure 3:**
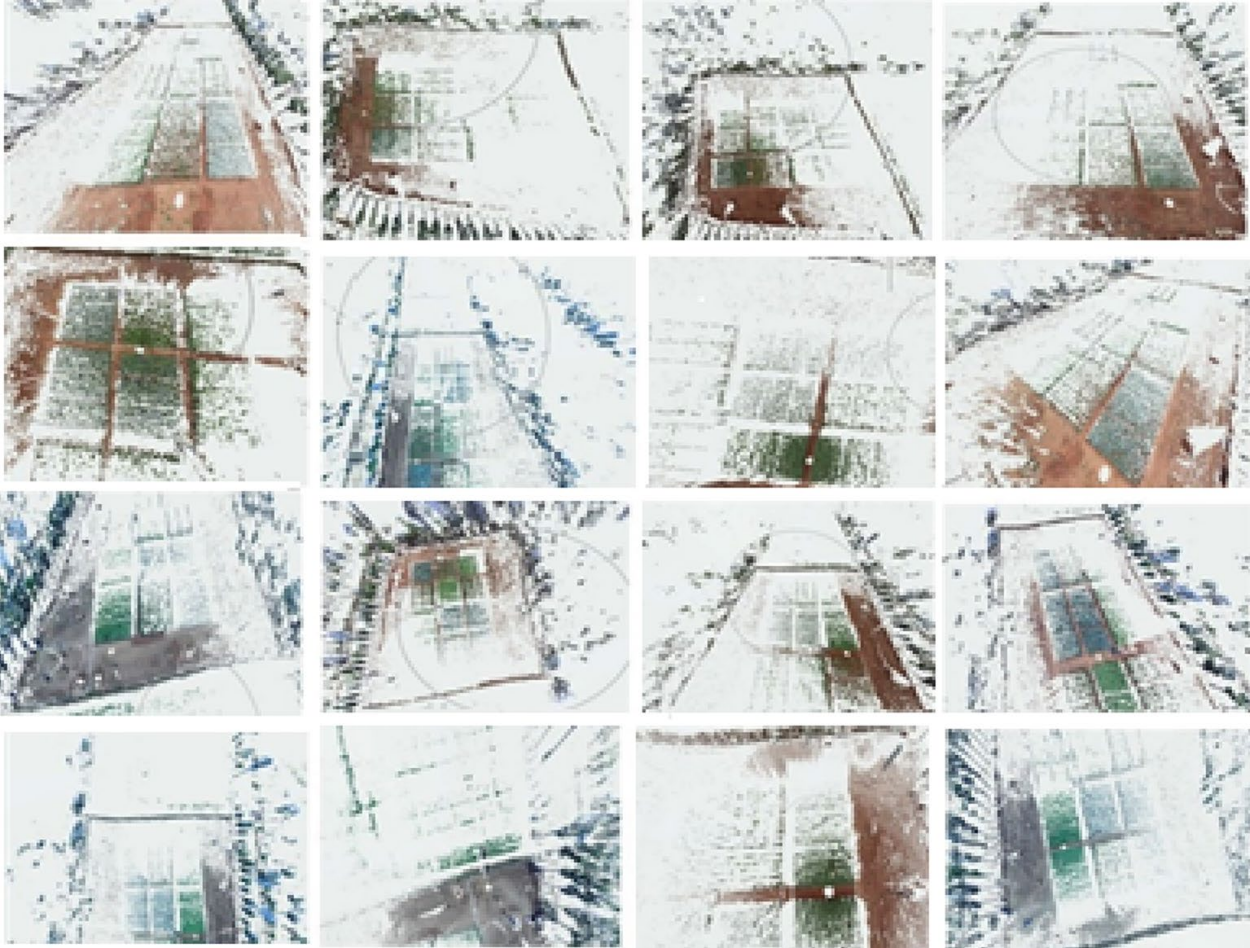
Scans of LiDAR point clouds from different positions in the experimental plots.

*LiDAR point cloud acquisition:* LiDAR point cloud was acquired for five different sampling dates using a 3D terrestrial laser scanner. The crops were sown on 27 March 2017, and subsequent sampling dates were selected at intervals to capture key growth stages and changes in crop structure. Specifically, the sampling dates were scheduled for 18 April, 4 May, 22 May, 14 June of 2017 to encompass various stages of plant development, from early growth to maturity, allowing for comprehensive data collection and analysis across different growth phases. On the first sampling date, a Riegl TLS (Model: VZ-400; Make: Riegl Laser Measurement Systems GmbH, Austria) was used. For the remaining three sampling dates, the TLS used was that of FARO (FARO 350^S^, FARO Technologies Inc., USA). Though the instruments were of different make, the scan density and the associated scan parameters were similar, and the resulting point cloud was seamless. The TLS scanner acquires laser returns in the electromagnetic spectrum's specified near-infrared wavelength (at 1550nm). The range of sensing is 350m. To cover the entire agricultural field, the TLS was placed at 16 different scan positions spread across different view directions in the experimental plots. Between each pair of successive scans, about 20–25% overlap was maintained to enable co-registration of the multiple scans of the point cloud. The tripod's height was adjusted to match the growth stage of the crops. To ensure acquiring a high-density point cloud, the scan duration was set to 10 min with a scan-point spacing of 6.1mm at 10m. To maintain point-level precision of geo-positioning of the point cloud, DGPS (Differential-GPS) measurements were acquired at each scan position, and the specified reference checkerboard targets were distributed in the plots. The same procedure was repeated for all the subsequent dates of point cloud acquisition. Figure 2 shows field photographs of a part of the experimental plots during datasets acquisition, and Fig. 3 depicts the acquisition of point clouds obtained from multiple angles.

### Methodology

**Figure 4:**
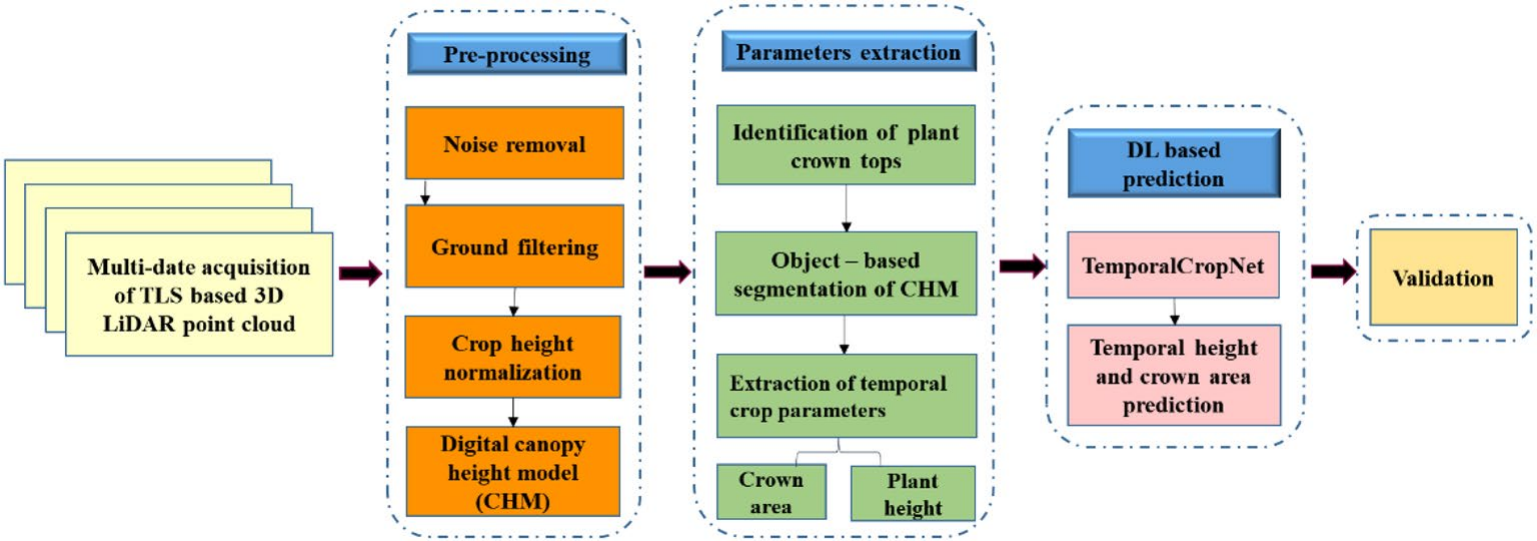
Flowchart depicting the steps involved in predicting crop parameters.

An overall view of the methodological process flow, broadly indicating the critical tasks and algorithms adapted for the realization of the goal of the work, is shown in Fig. 4.

#### Point cloud processing and crop canopy modelling

Due to the presence of plant support infrastructure and the possibility of multiple reflections from the nearby field boundary trees, some points could have out-of-the-context noise and outlier elevation values. We corrected the scans of point clouds by removing the noise and outlier removal by the neighbourhood thresholding method. Considering the DGPS-measured reference geo-coordinates of the sensor placement and checkerboard targets spread across the plots, the point clouds were co-registered using the multi-scan registration method suggested by^25^. To help maintain point-level matching across the scans and tide over the lack of sturdy corners in the point cloud of agriculture landscapes, tie points were established based on the guided sphere fit algorithm^26^. Each data file was geo-referenced to the World Geodetic System (WGS)-84 with UTM projection.

*Generation of crop height model (CHM):* As the structural parameters considered in this work pertain to the height and canopy of the plant, we classified the co-registered point cloud into the ground and above-ground point clouds. For this, we used the progressive triangular irregular network (TIN) densification (PTD) method^27^. To reduce the computational challenges, we divided the point cloud into several tiles. The reference surface was constructed based on a triangular irregular network (TIN) built using the ground points identified in each tile of the point cloud. The unclassified points were added to the TIN triangles based on the criteria of minimum distance to the TIN facet and the angle between the TIN facet and the line joining the point with the closest vertex. Continuing the triangulation in an iterative manner, the point cloud was classified into two categories: ground or above-ground. To compare the elevation of the point cloud across different growth phases, the elevation of the point cloud was normalized to the field furnace level. The plant-level canopy height model (CHM) was generated by rasterization of the normalized above-ground point cloud.

*Crown area estimation:* Plant level crown area was estimated by identifying the crown top of each plant and the subsequent delineation of the crown area. The plant crown tops were identified using the variable window filter (VWF) method proposed by^28^. For each pixel in the CHM, which represents plant height, the plant crown top was identified based on the local maxima defined by the radius of the window defined adaptively. Based on the centroid of intensity distribution indicated by the outcome of the VWF, the plant level crown area was modelled based on the semi-supervised segmentation of the raster CHM using the Watershed segmentation algorithm^29^. The possibility of over-segmentation was minimized by removing the segments which do not have associated crown top values.

Following the retrieval of plant height using the developed canopy height model (CHM) and crown area estimation utilizing variable window filter (VWF) for the three sampling dates, these parameters were inputted into the TemporalCropNet architecture to predict crop height for the fourth sampling date. In the case of tomato, sampling of the first three dates was considered as tomato crop was harvested before 4 June 2017.

#### Deep learning-based prediction of plant height and crown area

Deep learning (DL) techniques for image processing and pattern recognition in computer vision have reached maturity for operational applications, and a host of commercial products are developed using the standard optical RGB imagery^30–32^. Several DL techniques have been experimentally applied on multispectral and hyperspectral imagery for several tasks, such as segmentation, classification, unmixing etc. Point clouds acquired from various platforms have been processed using DL techniques for object detection, semantic segmentation, object-based landscape modelling and visualization etc. Several DL-based standard point cloud processing models are available^12,33–35^. Among the wide variety of model architectures and networks reported in the literature, convolutional neural networks (CNNs) and Recurrent Neural Networks (RNNs) are widely used for processing and analyzing remote sensing data. While CNNs are best suitable for instance-based processing tasks such as classification, RNNs are superior for processing time-series data.

The primary DL architecture adopted in this work is a variant of the RNNs. Unlike the feed-forward neural networks, RNNs use their internal state memory to process the data sequences. Recent studies confirm that DL networks such as Long Short-Term Memory (LSTM) and Gated Recurrent Unit (GRU) overcome the drawbacks of RNNs, such as vanishing gradient or exploding gradient problems. Based on the observations from the preliminary assessment of the performance of the LSTM and GRU for predicting the crop structural parameters independently, we used a stacked representation of LSTM and GRU. Combining multiple LSTM layers leads to greater model complexity and high-level time dependency. Stacked LSTM provides output for each time stamp and not the single output for all time steps. Similar is the case of GRU. Thus, a hybrid hierarchical model generated by stacking the LSTM and GRU helps improve the projection of information in latent dimension space, giving better prediction results. The stacked model was then fed to an individual fully connected layer (FCN) and then combined and given to the final FCN, where the feature concatenation occurs and the parameters are predicted. For ease of reference, we name the deep learning model developed for this specific purpose as 'TemporalCropNet'.

**Figure 5:**
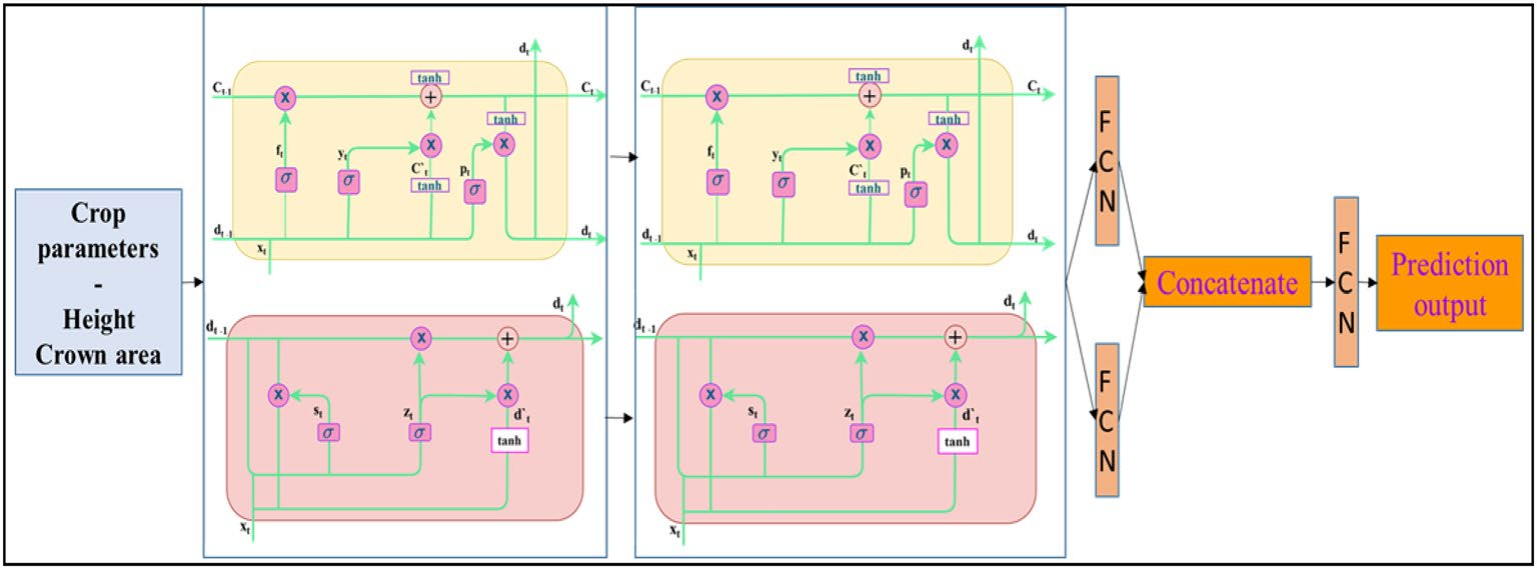
Stacked LSTM-GRU architecture of the proposed TemporalCropNet.

The top-level architecture of the TemporalCropNet is shown in Fig. 5. The crop parameters– height and crown area pertaining to different data acquisition dates are given as input to the TemporalCropNet. In summary, the hybrid hierarchical model of stacked LSTM and GRU layers, followed by fully connected layers, forms the core of TemporalCropNet, demonstrating significant improvement in predicting crop structural parameters. For ready reference, a brief description of the recurrent neural network (RNN), long short-term memory (LSTM), and gated recurrent unit (GRU) is given in Fig. 5.

*Recurrent neural networks (RNNs):* Unlike the feed-forward neural network such as CNN which has only a finite receptive field, a recurrent neural network (RNN) can process sequential data using the internal state memory. This dynamic nature of RNN finds application in audio, speech analysis and several other temporal-based applications. The recurrent state is achieved by connecting the time steps' output as input to the network and using the same weights to backpropagate through time. Therefore, RNN is a neural network that uses the shared weights for each time step. At the same time, RNN suffers from short-term memory when the sequence length becomes larger and suffers from vanishing gradient problems during backpropagation. Neural network weights are updated using gradients while backpropagating. The issue of vanishing gradient produced leads to minimal gradients and contributes nothing to the learning process, thus leading to short-term memory.

*Long Short-Term Memory (LSTM):* The cell state and gates are core structural elements of the sequential network, LSTM. A high-level representation of LSTM is similar to that of RNN but differs in terms of the internal representation of the memory cell. The input to the sequential network is a sequence of vectors (x_1_, x_2_,… x_n_), where x_t_ is the input at time t. The three basic units of the LSTM are forget gate (f_t_), input (y_t_), and output gate (p_t_). The forget gate determines to keep or discard the information from the previous time step. From the input gate, LSTM learns new information that enters the cell. The output gate passes the updated information in the cell from the present time step to the next. Similar to RNN, LSTM also has hidden state d_t_, d_t-1_, representing the current and next step's hidden unit. Also, the cell state is represented by c_t_ and c_t-1_ for the current and the next time-step, respectively. The cell state and hidden states are the two states of LSTM. All the gates undergo the sigmoid activation function, which gives the output value for each state and gate ranging between 0 and 1. In addition to that, LSTM also has a temporary cell state C'_t_, where the hyperbolic tangent activation function is applied that outputs values between -1 and + 1. In The final cell state C_t_, the forget gate f_t_ and input gate y_t_ determine how much information should be kept in the current time step. The new hidden state h_t_ for the next time step is determined by C_t_ and p_t,_ which determines the amount of information the memory cell has at the output step. For all cell states and gates, in addition to the weight parameter, bias value is also added to each equation from (1)—(5*).* As represented in Fig. 4, the hidden state dₜ and the cell state C_t_ for an LSTM cell can be calculated as follows.

1 $${y}_{t}=\sigma \left({x}_{t}{T}^{y}+ {h}_{t-1}{W}^{y}\right)$$

2 $${f}_{t}=\sigma \left({x}_{t}{T}^{f}+ {h}_{t-1}{W}^{f}\right)$$

3 $${p}_{t}=\sigma \left({x}_{t}{T}^{p}+ {h}_{t-1}{W}^{g}\right)$$

4 $$\tilde{C}_{t} = {\text{tanh}}(x_{t} T^{g} + d_{t - 1} W^{g} )$$

5 $${C}_{t}=\sigma ({f}_{t}* {C}_{t-1}+ {y}_{t}* {\widetilde{C}}_{t})$$

Here, y, f, p is called the input, forget, and output gates, respectively; W is the recurrent connection at the previously hidden layer and the current hidden layer, T is the weight matrix connecting the inputs to the current hidden layer. The GRU is an RNN architecture and is similar to LSTM units. The GRU comprises of the reset gate and the update gate instead of the input, output and forget gate of the LSTM. The reset gate determines how to combine the new input with the previous memory and analyses how much information is to be embedded with the current information. The update gate defines how much of the previous memory to keep around.

*Gated Recurrent Unit (GRU):* LSTM has a number of parameters which result in a number of operations to be performed. Thus, the variant of LSTM, GRU, is a simplified, compact representation of LSTM in terms of the parameters and operations performed. Unlike LSTM, GRU has only two gates: reset (s_t_) and update (u_t_), instead of the input, output, and forget gate of LSTM and a hidden state h_t_. The reset gate determines how to combine the new input with the previous memory, and the update gate defines how much of the previous memory to keep around. Also, the two gated combine the input x_t_ and d_t-1_ (information from previous state t-1). Since GRU has lesser tensor operations, it is faster to train the network than LSTM. The computation of z_t_, d_t_ is given in Eqs. (6) - (9) expressed below.

6 $${u}_{t}=\sigma \left({x}_{t}{T}^{u}+ {d}_{t-1}{W}^{u}\right)$$

7 $${s}_{t}=\sigma \left({x}_{t}{T}^{u}+ {d}_{t-1}{W}^{s}\right)$$

8 $${\widetilde{d}}_{t}=\text{tanh}({x}_{t} {T}^{d}+\left({s}_{t}* {d}_{t-1}{W}^{d}\right))$$

9 $${d}_{t}=(1- {u}_{t}* {d}_{t-1}+ {u}_{t}* {\widetilde{h}}_{t})$$

*The Stacking Mechanism*:

The stacked LSTM and GRU layers can be expressed as:

10 $${H}_{t}^{L}={LSTM}_{L}\left({H}_{t}^{L-1}\right)$$

11 $${H}_{t}^{G}={GRU}_{G}\left({H}_{t}^{G-1}\right)$$

where ${H}_{t}^{L}$ and ${H}_{t}^{G}$ in Eqs. 10 and 11 denote the hidden states of the LST and GRU layers at time t respectively, and L and G representes the number of stacked layers.

The outputs from the stacked LSTM and GRU layers are first fed into individual fully connected layers (FCNs). These individual FCN outputs are then concatenated and passed through a final FCN layer. Each stacked model's output H_t_ is processed through its respective FCN:

12 $${y}^{L}=\upphi \left({W}_{f}^{L} . {H}_{t}^{L}+ {b}_{f}^{L}\right)$$

13 $${y}^{G}=\upphi \left({W}_{f}^{G} . {H}_{t}^{G}+ {b}_{f}^{G}\right)$$

where ϕ is the activation function, ${W}_{f}^{L}$ and ${W}_{f}^{G}$ are the weight matrices, and ${b}_{f}^{L}$​ and ${b}_{f}^{G}$ are the bias vectors of the individual FCNs for LSTM and GRU, respectively in Eqs. 12 and 13.

The outputs ${y}^{L}$ and ${y}^{G}$ are concatenated and passed through the final FCN for prediction:

14 $$y_{concat} = \left[ {y^{L} ; y^{G} } \right]$$

15 $$\hat{y} = \phi (W_{final} . y_{concat} + b_{final} )$$

where $\widehat{y}$ is the predicted output, ${W}_{final}$ is the weight vector of the final FCN in Eqs. 14 and 15.

The training, test and validation datasets were taken as 60, 20, 20% of the total dataset. The predictions from the DL model were validated by comparing with the actual plant-level measurements. So as to ensure the robustness of the model to fit into unseen data, we have devised a fivefold cross validation strategy. The quality of the matching is represented by comparing the symmetric mean absolute percentage error (SMAPE) expressed in Eq. (16) ^36^.

Symmetric mean absolute percentage error,

16 $$SMAPE= \frac{100\text{\%}}{n}\sum_{i=1}^{n}\frac{\left|{y}_{i-}{x}_{i}\right|}{\left|{y}_{i}\right|+\left|{x}_{i}\right|}$$

Visualization of possible underestimation or overestimation relative to the one-to-one comparison of the measured and predicted values of the crop structural parameters is expressed using another graphical error metric, Logarithmic deviation^36^, given by

17 $$L_{d} = \mathop \sum \limits_{i = 1}^{n} log\left( {\frac{{y_{i} }}{xi}} \right),$$

where y_i_ is the estimated parameter, x_i_ is the measured parameter for the measurement pair.

### Ethical Approval

This study has not used data or samples pertaining to any humans or animals. No ethical committee approval is required for this study as such. This study has used data acquired on cultivated plants. We would like to declare that the study has complied with all the relevant guidelines applicable.

## Results and analysis

**Figure 6:**
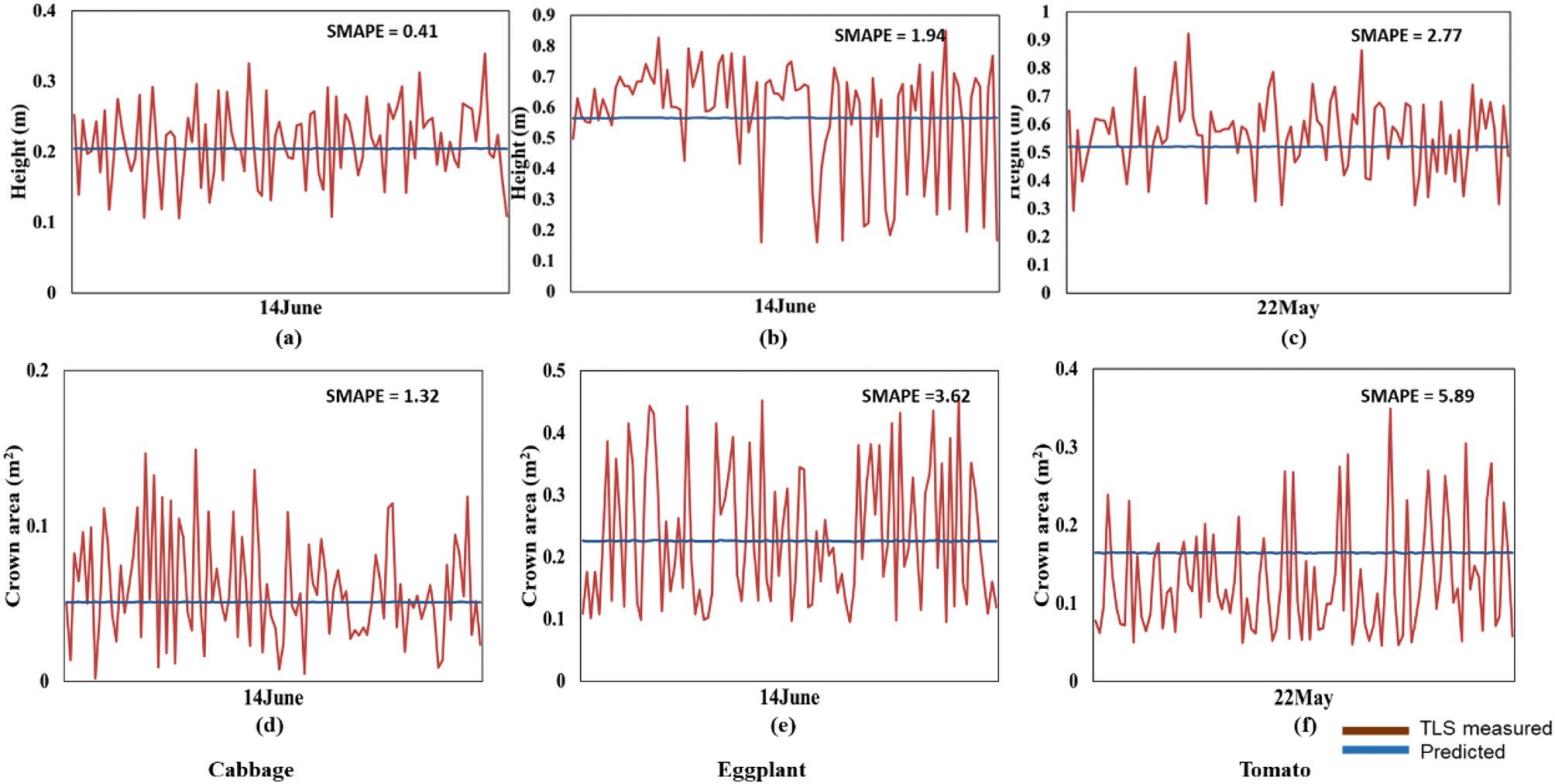
Predicted and TLS measured height and crown area plots for LSTM model the crops: cabbage (**a**,**d**), eggplant (**b**,**e**), and tomato (**c**,**f**).

The plant height and crown area were predicted using three different deep learning models. The first two types of predictions were obtained from the LSTM and GRU architectures independently, and the third model is the 'TemporalCropNet', the hybrid architecture of these two models. Results are presented for each of the three models. The temporal plant height and crown area prediction obtained from the LSTM model is shown in Fig. 6. As evident from Fig. 6, the prediction ability of LSTM is rather stagnant and indicates no response to the substantial variations in the structural parameters across different ranges and dates of the data acquisition. However, the major limitation is not the lack of predictive response to the training-induced learning capability of LSTM, but the magnitude of the structural parameter predicted. As illustrated in Fig. 6 for plant crown area, the LSTM model does predict the structural parameters considering the spatial variation of the corresponding across different growth stages. The quantity of predicted value is only one-half of the measured values, further calibrated with TLS estimates, especially the prediction of relatively lower values of the crown area or plant height is non-responsive from the LSTM model. This inconsistency in prediction is further corroborated by the logarithmic deviation metric computed for the prediction from the LSTM model, as shown in Fig. 6.

**Figure 7:**
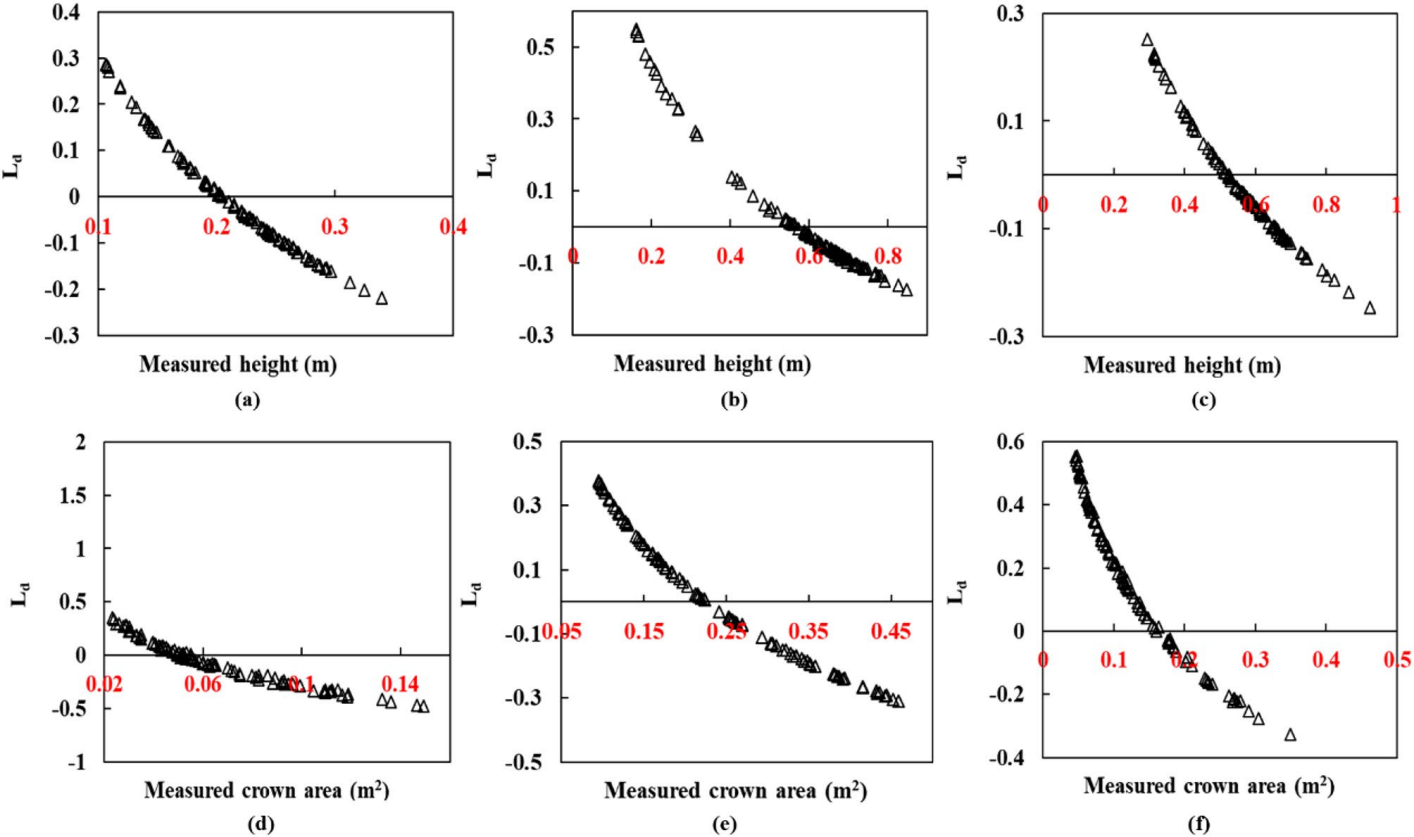
Logarithm deviation (L_d_) between the measured and predicted height (**a**–**c**), and crown areas (**d**–**f**) for the baseline model LSTM showing the over and underestimations in the prediction.

Figure 7 presents the logarithmic deviation of the height (Fig. 7a–c) and crown area (Fig. 7d–e) measurements derived from the LiDAR point cloud and predicted using LSTM. The direction of predicted values of the crown area or plant height indicated by L_d_ metric is rather monotonically decreasing, showing the lack of any responsive learning in the model prediction. The systematically looking distribution of points with a crossover of direction midway (Fig. 7) suggests that the predicted values are not in tune with the measured values of the structural parameters considered. For the predicted height in Fig. 7, even though there are numerous positive and negative deviations, most of the predictions are prone to negative estimation. Also, the extent of the deviation (L_d_) is mostly distributed from -0.2 to 0.2. Similarly, the log deviation depicts a considerable over and underestimation for the tomato crop. In the case of cabbage and eggplant, the number of samples which have undergone both estimations is mostly near L_d_ = 0, precisely between -0.1 and + 0.1. A contrasting pattern is visible in the case of the crown area. Here, many samples are in the direction of overestimation, and the extent of deviation is also larger than the height parameter.

**Figure 8:**
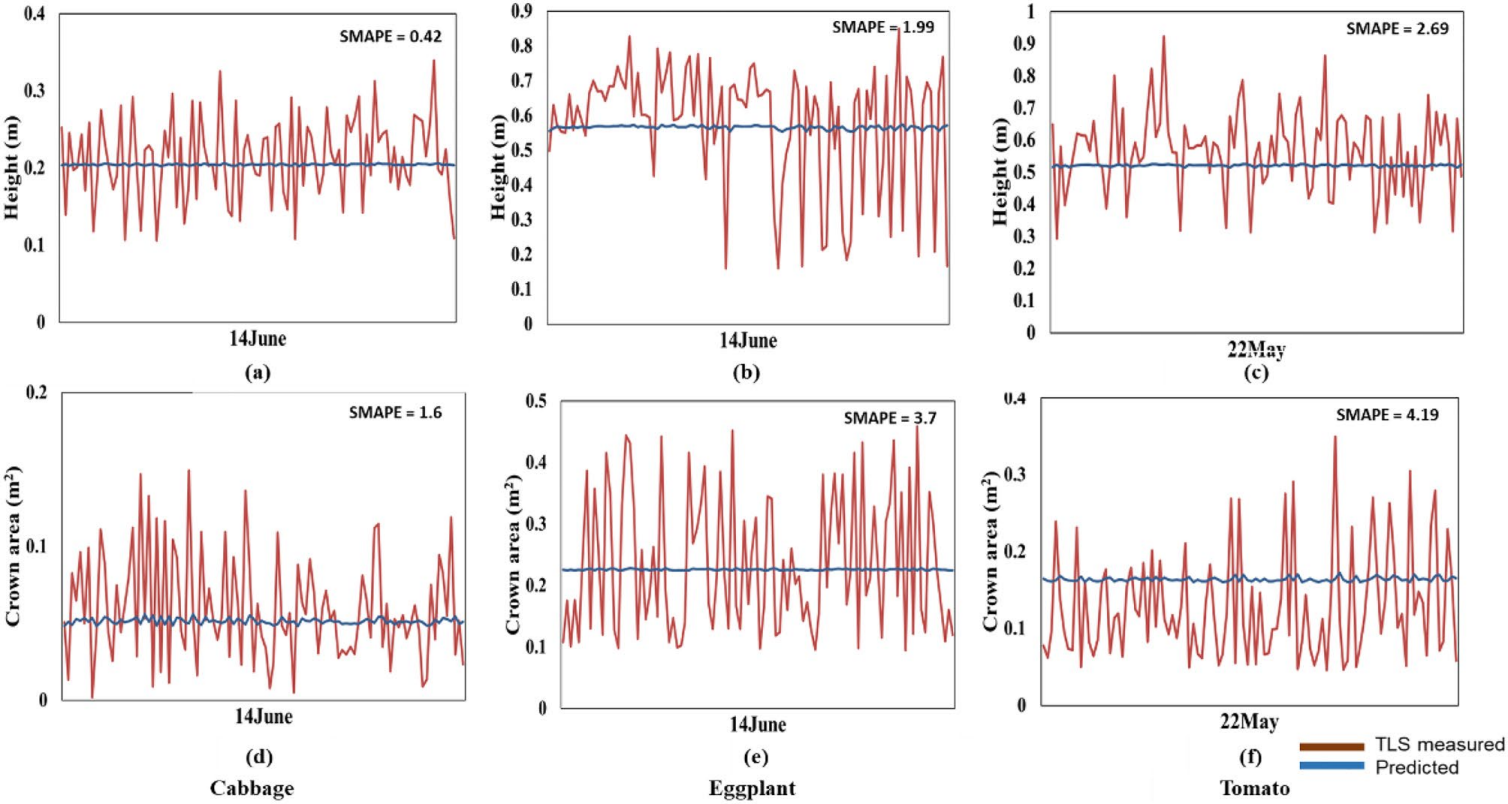
Predicted and TLS measured height and crown area plots for GRU model the crops: cabbage (**a**,**d**), eggplant (**b**,**e**), and tomato (**c**,**f**).

**Figure 9:**
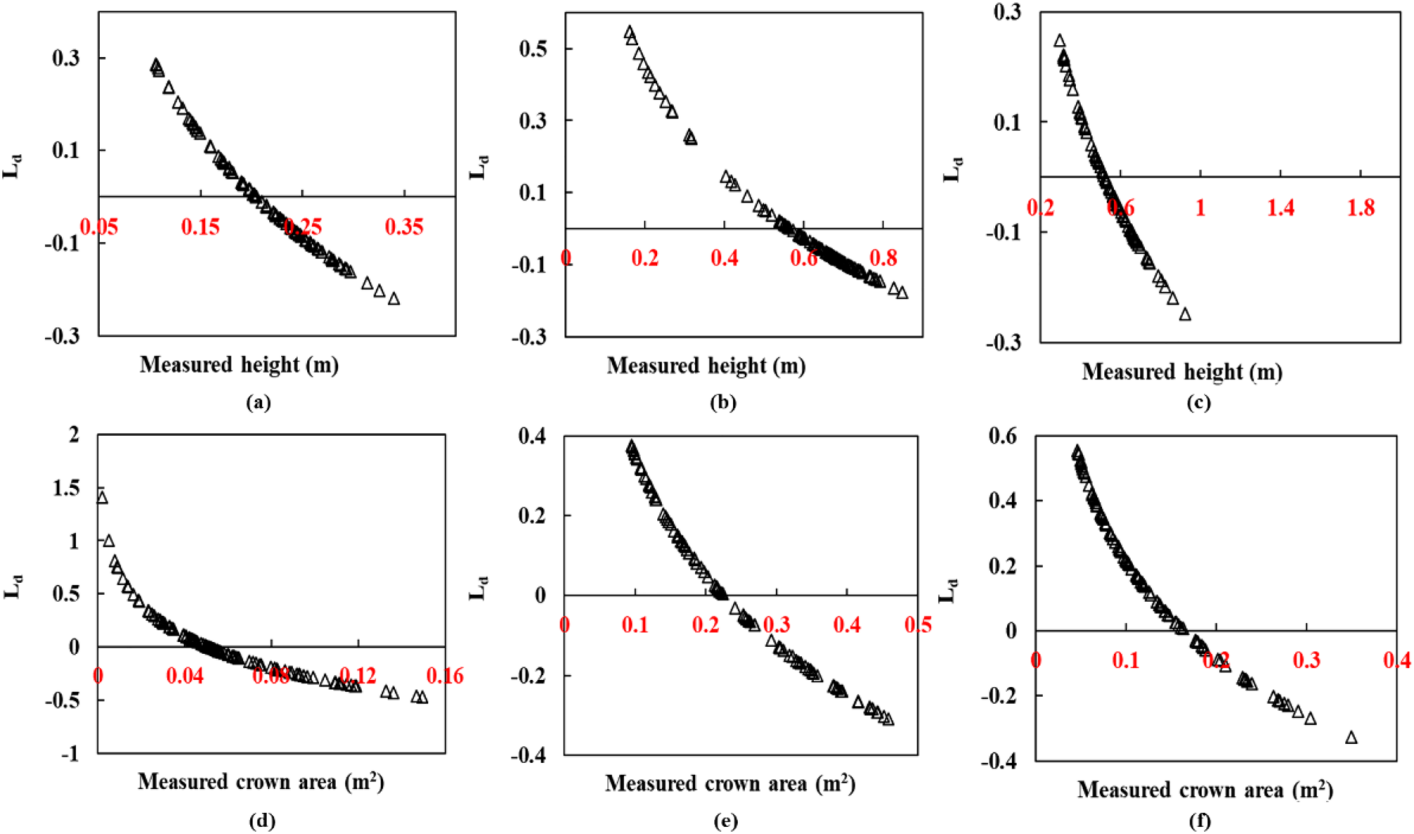
Logarithm deviation (L_d_) between the measured and predicted height (**a**–**c**), and crown areas (**d**–**f**) for the baseline model GRU showing the over and underestimations in the prediction.

**Figure 10:**
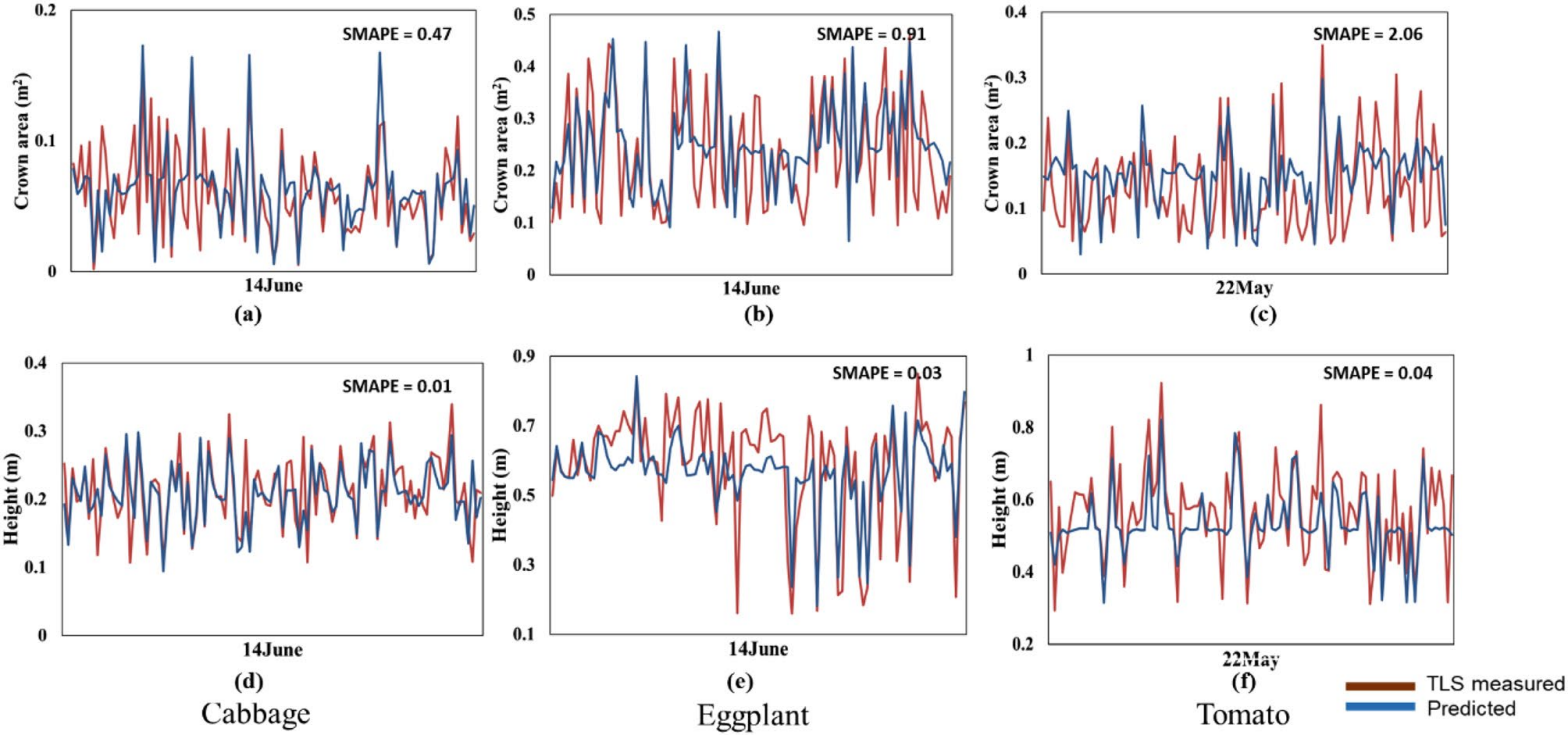
Measured and predicted plot of crown area and height for cabbage, eggplant and tomato for proposed TemporalCropNet.

The results of the prediction of the crop structural parameters from the GRU model are shown in Fig. 8. As seen in Fig. 8, the nature of predictions of the crop structural parameters for the GRU model closely follows the predictions from the LSTM model. At the overall level during the entire crop growth stage, the predictions are dynamic and in tune with the direction of variations of the measured values. However, the variations in the predicted values are limited by local gradients to the extent that most of the predictions at the lower values of the structural parameters are almost static. This lack of structural prediction is also evident in the values of L_d_ metric (Fig. 9). The complementary LSTM and GRU-based predictions are mainly in the change in the direction of predictions at different plant growth stages (Figs. 9 and 10).

**Figure 11:**
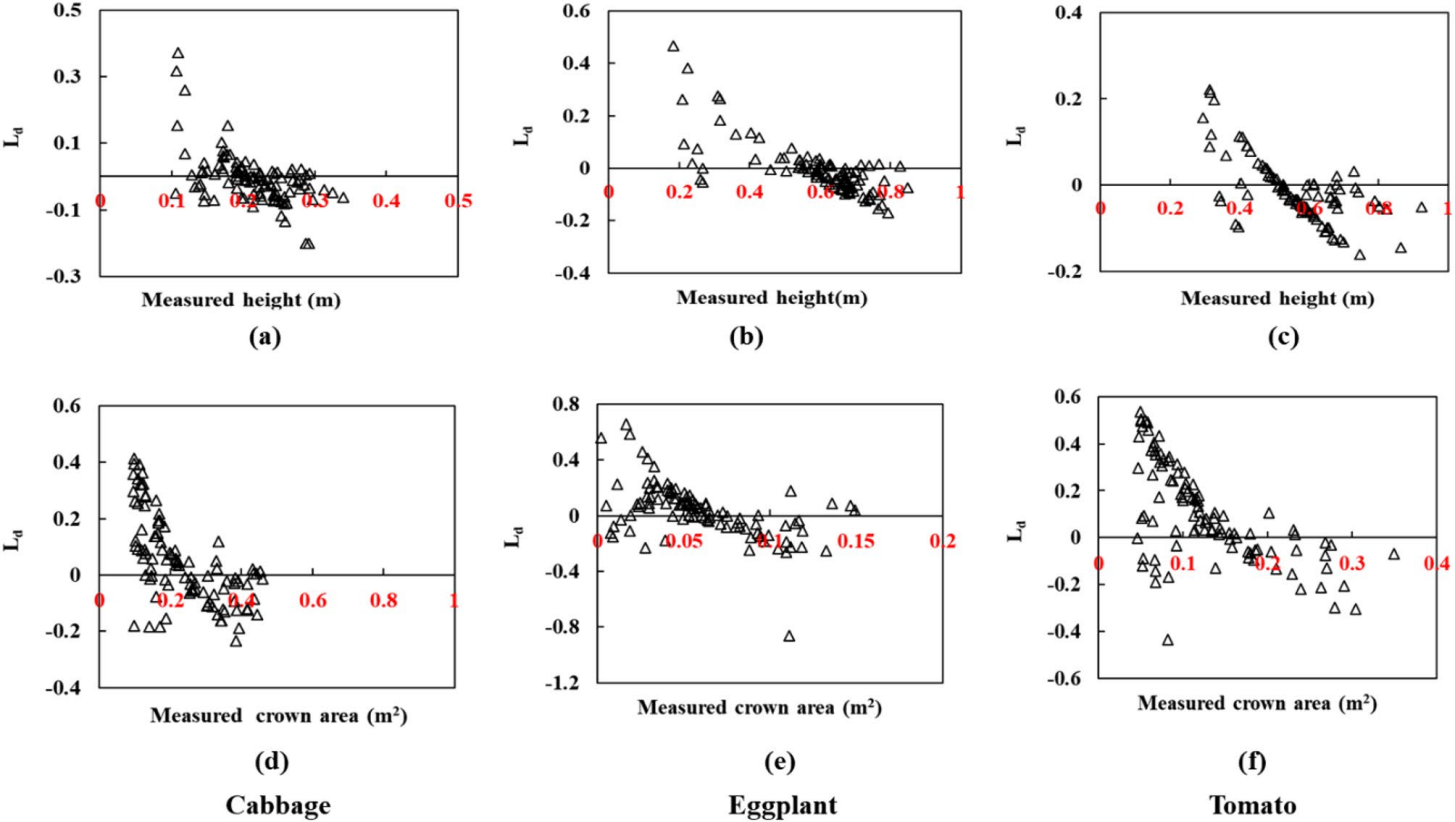
Logarithm deviation (L_d_) of the crown area showing the over and under-estimation of the crops for the proposed TemporalCropNet.

The results of the temporal prediction of structural parameters from the hybrid RNN model – TemporalCropNet are presented in Fig. 10. The directional variation of the predicted values vis-à-vis to the measured values are presented in Fig. 11. The predicted values of both the structural parameters considered are closer to the measured values across the growth stages, as indicated in Fig. 10. However, when the tomato crop is at the harvesting stage, the quality of prediction of the crown area is slightly lesser in precision compared to the measured values. The trend of the prediction across the crops is supported by the directional dependency of the predicted values as indicated by the L_d_ metric (Fig. 11). The predicted crown area and plant heights are slightly overestimated at the lower range of the measured values. The predictions are slightly underestimated at the upper ranges of the measured values. The predictions match almost one-to-one when the measured values are in the intermediate ranges. The variations of the predictions across different crops, as indicated by the SMAPE, show a slightly different pattern by the magnitude of the error. Converting the relative values of the estimate through the absolute percentage error, the error margin of the predicted values is between 5 to 12%. Comparing the performance of the TemporalCropNet with the results from LSTM or GRU, it can be summarized that the prediction of crop structural parameters is viable and depends upon the sophistication of the deep model and the inherent ability to derive the complementary nature of the two different types of architecture considered in this work. The correlation between the predicted and the TLS point cloud-based estimation of the plant height is shown in Fig. 10. The error assessment computed using SMAPE is 0.01 (Fig. 10d) for cabbage and 0.04 (Fig. 10f), which exhibits a minimal error rate for predicting crop height. However, for the crown area, the mean absolute error percentage is 0.4, 0.8 and 2 higher, respectively, for cabbage, eggplant, and tomato.

Interestingly, the same trend is visible for height and crown area prediction at different levels of N, where the error is calculated between the in-situ measurements and the LiDAR point cloud-derived parameters. Figure 11 shows the logarithmic deviation of the height (Fig. 11a–c) and crown area (Fig. 11d–e) measurements derived from the LiDAR point cloud and predicted using TemporalCropNet. For the predicted height in Fig. 11, even though there are numerous positive and negative deviations, more predictions are prone to negative estimation. Also, the extent of the deviation (L_d_) is mostly distributed from -0.2 to 0.2. Similarly, the log deviation depicts a considerable over and underestimation for the tomato crop. For cabbage and eggplant, the number of samples which have undergone both estimations is mostly near L_d_ = 0, precisely between -0.1 and + 0.1. A contrasting pattern is visible in the case of the crown area. Here, many samples are in the direction of overestimation, and the extent of deviation is also greater than the height parameter. Compared to Fig. 10 of the TemporalCropNet, L_d_ values are systematically over and underestimated in all six cases, showing the baseline LSTM and GRU models (Figs. 7, 9) in predicting the crown area. A similar trend is exhibited by the baseline models, LSTM and GRU, in predicting the height values (Figs. 7, 9). Likewise, a systematic positive and negative deviation is observed, which extends from 0.5 to 0.3.

**Figure 12:**
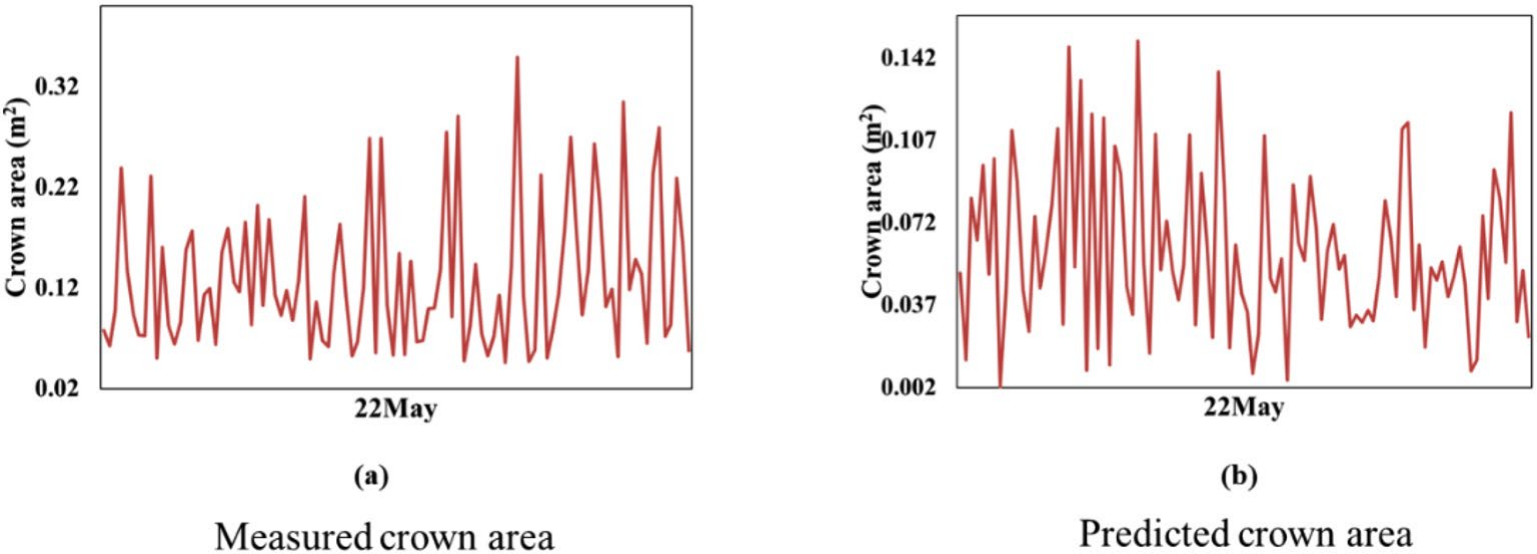
Variation of crown area values using baseline LSTM method.

Since the crown area value variation is negligibly small for the baseline models, a closer look at the measured and predicted crown area for LSTM is provided in Fig. 12. In this Fig., we can observe a visible height variation when the predicted value is plotted individually, as in Fig. 12b. However, the extent of the values for the predicted crown area is marginal. Hence, in Fig. 6, the predicted parameters appear as a line. This shows that both the baseline models fail to capture the inherent temporal growth pattern of the crops.

The prediction of temporal growth parameters such as height and crown area for horticulture crops has not yet been addressed in the literature, especially using LiDAR point cloud. This is a strong motivation for carrying out such studies and understanding the crop dynamics, temporal growth patterns and correlation among data, and subtle crop changes through that. Another important aspect of this study is to evaluate the temporal dependency within a field that follows intercrop farming practices. The concept of combining LSTM-GRU layers is the systematic approach that is followed for CNN models where several convolutional and fully connected layers are connected in a sequential manner for a better structure for various kinds of classification tasks that tend to derive non-linear temporal dependencies. We can embed several hidden layers for LSTM and GRU layers; in this experiment, the number of units of LSTM and GRU layers is set to 50. The number of epochs used for training is 400 epochs with a batch size of 50. A combined representative model of LSTM-GRU can learn the temporal dependency in the data in a much better way than the individual LSTM and GRU models. The baseline models failed to learn the crop growth pattern and the canopy area development as the crop grows and matures.

## Discussion

Providing spatial estimates of tangible or intangible parameters of different landscape elements (e.g. buildings, trees, crops, soils, water bodies, etc.) has been one of the primary applications of remote sensing data. Accurate prediction of plant height and crown area offers practical benefits in crop management, resource optimization, and yield estimation. By providing precise information on plant structure, farmers can tailor their management practices, such as irrigation and pest control, to optimize crop health and productivity. This, in turn, leads to more efficient resource allocation, reduced environmental impact, and improved sustainability in agriculture. Additionally, accurate predictions enable better yield estimation, aiding in production planning and marketing decisions while enhancing food security.

Estimation of biophysical parameters related to vegetation, referred to as biophysical characterization, has been extensively researched over the past three decades, as outlined in the literature review. Various statistical, machine learning, and emerging deep learning approaches have been extensively explored for this purpose. The general approach involves acquiring spatially distributed reference measurements at select locations within the study site covered by remote sensing data, followed by the development of classification or regression models to generate spatial maps of the parameters of interest. Increasingly, at the level of individual vegetation elements, particularly trees in urban and forest environments, efforts are being made to delineate and characterize structures using LiDAR point cloud data, often employing deep learning methods^37–39^. The successful generation of geographically extensive coverage maps of vegetation heights^40,41^ and tree crown area canopy affirm the promising combination of deep learning models and LiDAR point cloud. Apart from applying to a different domain of vegetable crops, exploring the potential of the combination of LiDAR point cloud and deep machine learning models for the estimation of plant-level structural parameters at different growth stages is a distinct feature of research carried out in this thesis. Given the limitations and functional constraints of different deep machine learning methods, the generation of hybrid models broadly within the context of ensemble modelling has been considered a viable option. Broadly related to this perspective, the application of two different deep machine learning models – LSTM and GRU, and the hybrid model generated, 'TemporalCropNet' is aimed at expanding the horizon of the single state' estimation' of biophysical parameters to 'prediction' thereby enabling the predictive maps of crop growth profiles for advanced crop management.

The performance of 'predicted' values of two important crop structural parameters – plant height and the crown area has been compared with the ground truth reference values measured throughout the crop growing season. Analyzing the results obtained from the base models (LSTM and GRU) and the proposed hybrid model 'TemporalCropNet' suggests crucial insights. The quality of the predictions of structural parameters from the LSTM is poor because the magnitude of the prediction is less than one-half of the actual value. Further, the LSTM model is non-responsive to the variation of structural parameters both at the lower and higher-level values. While following the general trend of the predictions from the LSTM, results from the GRU suggest the exitance of a stepped gradient in the predictions, indicating the dominance of local features in the predictions of GRU. As a result, the point-on-point plot of structural parameters (Figs. 5 and 7) of the predicted with the LiDAR point cloud-based values show a limited extent of the predicted values by amplitude and direction. The proposed 'TemporalCropNet' has exploited the complementary functional performance of the LSTM and GRU, thereby offering 'predictions' of crop structural parameters at the plant level closely matching with the TLS LiDAR point cloud-based values. The 'TemporalCropNet' is developed and implemented to predict plant-level height and crown area. As the model is based on an open architecture with the flexibility to ingest different ranges of values, the method can be extended to predict crops' other biophysical parameters.

It is crucial to address the various factors that scould affect the effectiveness and applicability of the proposed approach. The data collection stage can be challenging due to various environmental factors, such as wind and rain, which might lead to inconsistencies in the collected data. Additionally, the generalization of the developed model across different crop types or environmental conditions is a significant concern. Crop morphology, growth patterns, and environmental factors vary widely, and the model's performance may be compromised when applied to standing crops with different characteristics than those in the training data.Incorporating data from multiple sensors alongside LiDAR can enhance the predictive capacity of models for crop height and crown area. This approach enables a more comprehensive understanding of crop characteristics and facilitates accurate predictions. Future research can focus on developing integrated models that leverage diverse sensing technologies to improve agricultural monitoring and management.

## Conclusions

Based on the crop plant's structural attributes at past growth stages, prediction of the future structural parameters of the crops at the crop patch/plant level has been attempted first time, to the best of our literature review. To achieve this, a deep learning-based model named 'TemporalCropNet', integrating LSTM and GRU, has been developed for individual plant-level prediction. The structural parameters are derived from the CHM developed from the LiDAR point cloud during the crops' key phenological growth stages. The predicted values are compared with the actual LiDAR point cloud-derived values Our novel methodology involved deriving structural parameters from LiDAR point cloud data, specifically the canopy height model (CHM) and crown area, acquired during key phenological growth stages of tomato, eggplant, and cabbage crops. The predictive accuracy of 'TemporalCropNet' was rigorously evaluated and compared against baseline LSTM and GRU models, as well as TLS-derived estimates. Our findings demonstrate that 'TemporalCropNet' outperformed the baseline models, exhibiting significantly lower error rates, particularly in predicting plant height. Furthermore, our analysis revealed the robustness of the model in capturing temporal dependencies, thereby enabling the ex-ante prediction of crop structural parameters. This capability holds immense potential for developing dynamic farming management practices, allowing agricultural practitioners to make timely and informed decisions throughout the crop growth cycle at a temporal scale. Future studies could focus on expanding the model's capabilities to predict advanced growth stages based on single-stage crop status. By improving the model's ability to predict accurately plant growth traits and handle larger datasets, we can discover new ways to apply precision agriculture, which will help sustain farming practices and ensure food security.

## Data Availability

The authors will be glad to share the data used in this work for any academic researchers free of charge. We encourage the interested researchers to contact the corresponding author (E-mail: rao@iist.ac.in).
